# An Optimized Node Deployment Solution Based on a Virtual Spring Force Algorithm for Wireless Sensor Network Applications

**DOI:** 10.3390/s19081817

**Published:** 2019-04-16

**Authors:** Xiaohua Deng, Zhiyong Yu, Rongxin Tang, Xin Qian, Kai Yuan, Shiyun Liu

**Affiliations:** 1 Institute of Space Science and Technology, Nanchang University, Nanchang 330031, China; dengxhua@gmail.com (X.D.); spacelab.ncu@gmail.com (Z.Y.); y_k_phy@hotmail.com (K.Y.); 2 Lunar and Planetary Science Laboratory, Macau University of Science and Technology, Macau 999078, China; 3 One Microsoft Way, Redmond, WA 98052-6399, USA; chianshin@gmail.com; 4 Usun Microelectronics, Nanchang 330072, Jiangxi, China; mackliu@usunmicro.com

**Keywords:** wireless sensor network, virtual spring force, hexagonal topology, pair correlation diversion, energy consumption

## Abstract

How to effectively deploy all wireless sensors and save a system’s energy consumption is a key issue in current wireless sensor network (WSN) applications. Theoretical analysis has proven that a hexagonal structure is the best topology in the two-dimensional network, which can provide the maximum coverage area with the minimum number of sensor nodes and minimum energy consumption. Recently, many scientists presented their self-deployment strategies based on different virtual forces and discussed the corresponding efficiency via several case studies. However, according to our statistical analysis, some virtual force algorithms, e.g., virtual spring force, can still cause holes or twisted structure in a small region of the final network distribution, which cannot achieve the ideal network topology and will waste the system energy in real applications. In this paper, we first statistically analyzed the convergence and deployment effect of the virtual spring force algorithm to derive our question. Then we presented an optimized strategy that sensor deployment begins from the center of the target region by adding an external central force. At the early stage, the external force will be added to the most peripheral nodes to promote the formation of hexagonal topology and avoid covering holes or unusual structure. Finally, a series of independent simulation experiments and corresponding statistical results proved that our optimized deployment solution is very stable and effective, which can improve the energy consumption of the whole sensor network and be used in the application of a large scale WSN.

## 1. Introduction

With the rapid development of 5G and IoT technologies, wireless sensor networks get much more attention in our life and have real applications in many fields, such as industrial robot, environmental exploration, military monitoring and human health etc. [1,2,3,4]. However, in some dangerous or inconvenient environments, like underwater or disaster areas, it is very necessary for self-deployment solution to satisfy the requirement of precise measurement. As for a wireless sensor network, how to effectively readjust sensor locations via self-deployment strategy to achieve an ideal coverage with minimum sensor nodes has already become a hot topic in recent years [5]. Many scientists focused on it in two-dimensional plane or three-dimensional space environment applications [6,7].

Generally, the well-accepted optimal topology of a two-dimensional sensor network is regular hexagonal topology but the best topology of a three-dimensional network is still under discussion. Here we mainly focus on the two-dimensional sensor network deployment. Previous researches on 2D self-deployment can be classified into three categories: the methods based on virtual physics model, the methods based on computational geometry and the methods based on biogeography. In terms of the virtual physics model strategy [8,9], it looks the sensor node as an electron or molecule so that all nodes move toward to each other by their virtual forces or potential fields. However, the topology of their overall network is relatively dense in the center and is sparse in the edge due to some physical reasons. According to the computational geometry-based approach [10,11,12,13], nodes readjust their positions by virtual force generated from adjacent nodes which satisfy a certain geometric condition. As for the method based on biogeography [14,15,16], nodes readjust their positions by simulating biological habits. It is also hard to achieve ideal hexagonal network.

However, in previous virtual force algorithm papers, most of the simulations verifying their deployment methods have been single and not universal [8,9,10,11,16,17]. For example, based on our retests in virtual exchange force [10,11], spring force [17], Van der Waals forces [8], and dusty plasma crystallization [9] algorithms, the experiments may have different deployment effects due to different initial sensor network even using the same simulation parameters. Therefore, in this paper, this is the first time to use a large number of experiments to statistically study the validation and stability of virtual force algorithm.

Here, we choose a virtual spring force algorithm which belongs to the above second category to obtain the optimization strategy since it has low complexity and fast convergence rate. Most of the previous self-deployment works based on the virtual force algorithm have a little bit imperfect region that includes coverage redundancy or omission [9,11,17]. This is a common case in many deployment solutions [8,10]. It is not difficult to understand that all sensor nodes are moving at the same time, which may cause the sensors in the outer region to reach a stable topology first. However, the number of sensor nodes in the inner region is not exactly the same with the hexagonal topology and has to form some twisted structure or leave a hole there. Our statistical analysis clearly demonstrates this disadvantage and instability.

In order to solve the above problem, we adopt an optimization solution that node deployment begins from a center region by adding an external force to the most peripheral nodes of the network. Other sensor nodes are gradually involved to promote the perfect formation of hexagonal topology. It turns out that the shift from global computation and deployment to centrally preferred deployment is very stable and effective. Further statistical analysis also showed that all the wireless sensor networks, no matter what the initial distribution is, finally yield perfect hexagonal topology. The optimized redeployment can work for non-standard deployment cases. In addition, the calculated moving distance indicates that this new optimization algorithm yields lower energy consumption than the original virtual spring force algorithm.

Node location will affect the calculations of node velocity and acceleration so that location accuracy is important in common virtual force algorithms. Our optimized method is a valid algorithm which presents a possible node deployment strategy in WSN applications, which theoretically provides a hexagonal structure in the two-dimensional network with the maximum coverage area and the minimum number of sensor nodes. As the rapid development of unmanned aerial vehicle (UAV) in environment monitoring or space exploration, such us desert, deep space or deep sea applications etc., the high accuracy of node location may become important in the near future since a large-scale WSN can save many resources based on the precise node deployment. Furthermore, our algorithm can provide a perfect hexagonal topology which can be used to improve other self-deployment methods and achieve a hybrid optimization.

The rest of this paper is organized as follows. Section 2 describes the fundamental theory of virtual spring force algorithm. Section 3 gives the system performance evaluation based on pair correlation diversion method, the simulation environment and scenarios. Then it presents main numerical parameters, detailed simulation results, statistical analysis of 100 independent experiments to prove the validation of VFA-SF and to demonstrate its disadvantage and instability. Furthermore, our optimization strategy (VFA-SF-OPT) is shown in Section 4. We retested all 100 experiments and the final node distributions are perfect hexagonal network topology. In addition, the comparison of system energy consumption also indicates that VFA-SF-OPT is better than the original VFA-SF. Conclusions are summarized in Section 5.

## 2. Virtual Force Algorithm Inspired by Spring Force

Virtual force algorithm is one of the most popular mechanisms for node deployment in wireless sensor network applications, in which sensors move by the virtual force determined by the relative position of neighboring nodes and the presence of an event. In this section, we present an algorithm inspired by virtual spring force (VFA-SF).

It is assumed that all sensors have identical capabilities of sensing, communication, computation and mobility. A sensor can communicate with any other sensors within the communication range $R_{c}$. And they can monitor the region and collect data within the sensing range $R_{s}$. If the sensor network have been deployed to the perfect hexagon topology, the distance between two neighbor nodes becomes $D_{m}=\sqrt{3}R_{s}$. In order to communicate with the neighbor nodes, the communication range of every sensor must be greater than $\sqrt{3}R_{s}$. Furthermore, each sensor is capable of moving by itself and detecting its own location by some method (e.g., the GPS, or iterative multilateration [18]).

Then, at the beginning, all *N* nodes in a wireless sensor network were randomly deployed in the target region whose area is *S* and the center is located at (0,0). The location of each sensor $n_{i}$(i=0,1,...,N-1) is given by a vector ${\vec{x}}_{i}\left(t\right)$ (${\vec{x}}_{i}\left(t\right)$ also indicates the position of node *i* at time *t*). The vector ${\vec{d}}_{ij}\left(t\right)$ indicates the distance between node *i* and node *j* at time *t*.

The spring force was chosen as the core of the “virtual force” [18]. The force on node *i* at time *t* is driven by the Newton motion law:

(1) $$m\frac{d^{2}{\vec{x}}_{i}\left(t\right)}{dt^{2}}={\vec{F}}_{i}\left(t\right)$$

where *m* is the mass of a sensor node and ${\vec{F}}_{i}\left(t\right)$ is the resultant force. ${\vec{F}}_{i}\left(t\right)$ can be defined as the sum of three components:

(2) $${\vec{F}}_{i}\left(t\right)={\vec{F}}_{i}^{e}\left(t\right)+{\vec{F}}_{i}^{f}\left(t\right)+{\vec{F}}_{i}^{ce}\left(t\right)$$

where ${\vec{F}}_{i}^{e}\left(t\right)$ is the virtual spring force, ${\vec{F}}_{i}^{f}\left(t\right)$ is the friction force (also call it damping force), and ${\vec{F}}_{i}^{ce}\left(t\right)$ is the centripetal force.

Generally, spring forces are established between pairs of sensor nodes if one node has no any other nodes in the upper and lower 60 degree sectors with another node and their distance is smaller than $R_{c}$. Here, we use ${\vec{x}}_{ij}\left(t\right)=\left({\vec{x}}_{j}\left(t\right)-{\vec{x}}_{i}\left(t\right)\right)/d_{ij}\left(t\right)$ to calculate the normalized vector from node *i* to *j*. If the set of possible node *j* acting with virtual forces on node *i* ($d_{ij}\left(t\right)<R_{c})$ is expressed by $\Phi_{i}\left(t\right)$, the corresponding total spring force of node *i* is

(3) $${\vec{F}}_{i}^{e}\left(t\right)=\sum_{j\in \Phi_{i}\left(t\right)}\kappa \left(d_{ij}\left(t\right)-D_{m}\right){\vec{x}}_{ij}\left(t\right)$$

where κ is a spring coefficient.

The damping force ${\vec{F}}_{i}^{f}\left(t\right)$ is defined as

(4) $${\vec{F}}_{i}^{f}\left(t\right)=\left\{\begin{array}{c} -\min\left(\gamma_{0}{\vec{x}}_{0}\left(t\right),\;{\vec{F}}_{i}^{e}\left(t\right)+{\vec{F}}_{i}^{e\upsilon }\left(t\right)\right),\;\;\mathrm{when}\;\;d{\vec{x}}_{0}\left(t\right)/dt=0\\ -\gamma d{\vec{x}}_{i}\left(t\right)/dt,\;\;\;\mathrm{otherwise} \end{array}\right..$$

This damping force is to reduce the elastic potential energy of the whole network and accelerate the convergence of the node deployment so that the damping parameter γ has great influence on the deployment solution. Once the damping of spring oscillator is absent, the oscillator should work in a simple harmonic vibration state, and $\omega_{0}$ is the natural vibration frequency. However, in order to deploy a WSN system quickly to an equilibrium state with small systematic energy consumption, a suitable damping parameter must be found.

**Figure 1:**
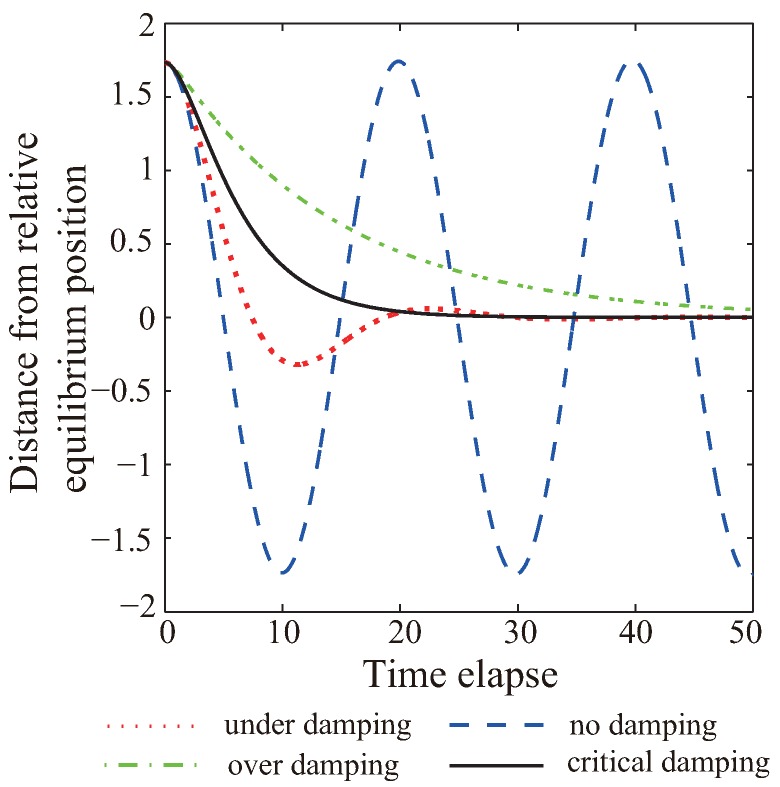
Spring vibration under different damping.

Let $\kappa /m=\omega_{0}^{2}$ and γ/m=2β where β is the real vibration frequency and related to the damping effect. Usually, larger β corresponds to a larger damping force. A new quantity ε is defined as $\varepsilon =\gamma /(2\sqrt{\kappa m})$. Figure 1 shows the vibration of the spring movement under different damping conditions. If the spring oscillator has no damping, it would work in a simple harmonic vibration state (dashed blue). The red dotted line and the dashed green line present the spring oscillator under damping and over damping, respectively. When ε=1 and $\beta =\omega_{0}$, the sensor network operates in critical damping condition (solid black). The elastic potential energy is decreasing by the damping force and the system would converge quickly to an equilibrium state without unnecessary vibration or energy consumption. Under this circumstance, the value of parameter γ is theoretically optimal and $\gamma =2\sqrt{\kappa m}$.

The centripetal forces is defined as

(5) $${\vec{F}}_{i}^{ce}\left(t\right)=-F_{centri}\times {\vec{x}}_{i}\left(t\right)$$

which is only an external auxiliary force added on each sensor node. With the centripetal force, sensors will move closer to the region center and always return to the sensor networks. It has an inward transition effect, which means one inner sensor node is subject to the sum of its own centripetal force and the centripetal force of the sensor nodes outside the sensor network. Therefore, the $F_{centri}$ constant must be much smaller than the spring force so that it will not affect the main virtual algorithm. Once a wireless sensor networks has been deployed to the final hexagon topology, this force would be released in the algorithm.

## 3. Simulations and Statistical Analysis for VFA-SF

### 3.1. Performance Evaluation of the VFA-SF

In the present study, a novel performance metric based on the pair correlation function within the crystalline structure was introduced to evaluate the topology of the sensor network. The pair correlation function g(r)=(N(r,Δ)S)/(2πrΔN) within the network structure represents the probability of finding two nodes separated by a distance *r* where *S* is the area of coverage, *N* is the number of sensor nodes contained in *S*, and N(r,Δ) indicates the number of nodes located between r-Δ/2 and r+Δ/2. Therefore, the pair correlation diversion is

(6) $$\delta \left(\Omega ,\Omega_{H}\right)=\frac{\int_{0}^{r_{T}}{\left\|g_{\Omega }\left(r\right)-g_{\Omega_{H}}\left(r\right)\right\|}^{2}dr}{\int_{0}^{r_{T}}{\left\|g_{\Omega_{H}}\left(r\right)\right\|}^{2}dr}\;,$$

where Ω is the network topology to be analyzed, $\Omega_{H}$ is the perfect hexagonal topology, and $r_{T}$ is the bound of the radial distance.

The pair correlation diversion (PCD) provides an objective metric for evaluating virtual-force algorithms. It quantitatively analyzes the proximity from any network topology to a perfect hexagonal configuration [9,11]. This metric can indicates the uniformity of the sensor network. Moreover, it can also be used to characterize the convergence rate of any virtual-force algorithm. The slope of the pair correlation function curve, as a function of the simulation steps, indicates how fast the virtual force algorithm converges.

For a wireless sensor network, when the value of $\delta (\Omega ,\Omega_{H})$ is small, the network coverage topology approximates a hexagonal structure. Generally, a perfect deployment solution will have a PCD value of 0. In addition, the convergence rate can be obtained according to the decrease of PCD curve. When a network reaches its steady state, the PCD value should also become stable.

### 3.2. Simulation Descriptions of the VFA-SF

We set the number of sensor nodes N=500 in the target region. Therefore, the coverage area by each sensor as a hexagon cell is $S_{hexagon}=\left(3\sqrt{3}\right)/2R_{s}^{2}$ and the total coverage area of the perfect hexagon topology is $S_{perf}=N\times \left(3\sqrt{3}\right)/2R_{s}^{2}$.

The mass of each sensor is normalized to m=1 since the sensor can be seen as a particle without considering the hardware design and mechanical parts in this algorithm. The sensing radius $R_{s}=1$ which is a normalized radius depending on different wireless sensor. If the real sensing radius of one sensor node is greater, the final coverage of the whole wireless sensor network is larger. We set spring coefficient κ=15 and $F_{centr}=0.005$, which corresponds to $D_{m}=\sqrt{3}$, $\gamma =\sqrt{4\kappa m}=7.75$.

The communication radius $R_{c}$ is also a normalized value. Generally, the communication range of one sensor is limited, but it is necessary to ensure that the adjacent sensor nodes can communicate with each other. Based on the topology of a perfect hexagonal network, the distance between two adjacent nodes is $\sqrt{3}R_{s}$. $R_{c}$ must be larger than $\sqrt{3}R_{s}$. Furthermore, the communication radius can be used to shield the nodes with a longer distance in the virtual force algorithm. Only the nodes inside the $R_{c}$ will be involved in the VFA-SF algorithm. In this paper, we set $R_{c}=3R_{s}=3$ which means only the spring forces from closest neighbor nodes are calculated for the total force, acceleration, velocity, etc. We think this a reasonable ratio which reader can find the appropriate wireless sensor for real field test.

Here are the detailed process of the VFA-SF:

(1) Initially, the 500 sensor nodes were randomly scattered in a circular area;
(2) By selecting adjacent nodes through hexagonal relation and shielding distance $R_{c}=3$, virtual spring force will be generated between neighboring nodes.
(3) Perform the following steps for all nodes in a loop: (a) calculate the sum of all virtual spring forces of node *i*; add resistance and centripetal force, and calculate the acceleration ${\vec{a}}_{i}$ of node *i* at time *t* according to the Newton’s motion equation; (b) The velocity ${\vec{v}}_{i}$ and position coordinate ${\vec{r}}_{i}$ of node *i* at t+dt are obtained.
(4) Repeat (2) and (3), and update the coordinate, speed and acceleration information of each node every dt time step until the whole network is relatively stable.

In this paper, we used a computer with i7-6700@3.4GHz CPU and 8G memory and run our code in Microsoft Visual C++ 2012 to achieve the virtual force calculation. Corresponding time step in the simulation tools is dt=0.08 s. Then the PCD performance in a circular area with a radius of 19 was evaluated every 5 time steps. It includes almost all sensor nodes and is very convincing to evaluate the overall network topology. The total calculation time (5000 time steps) for a 500 nodes network only needs about 45 s.

### 3.3. Statistical Analysis for VFA-SF

**Figure 2:**
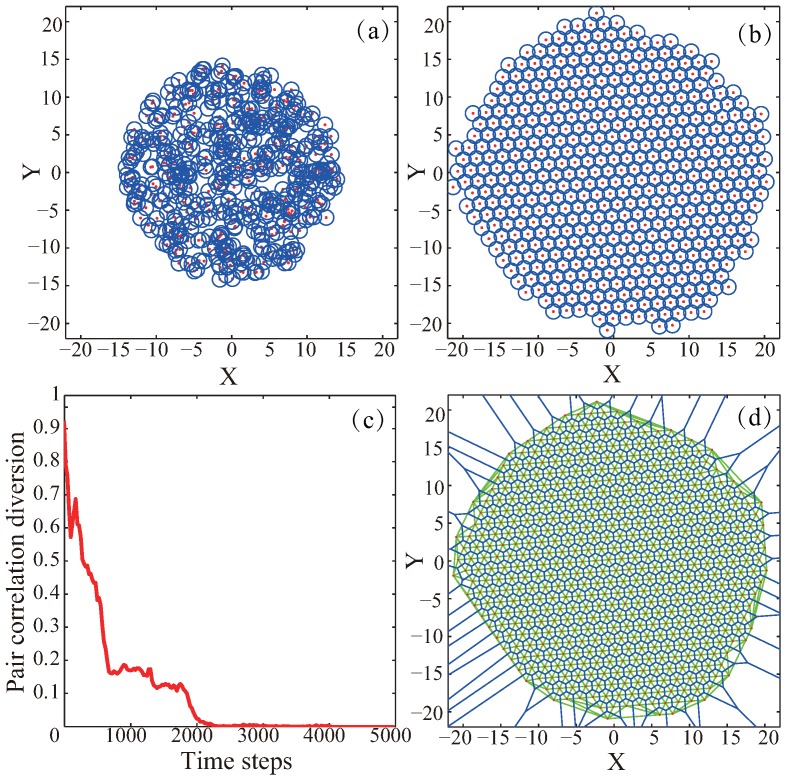
(**a**) Initial node distribution; (**b**) Final node distribution; (**c**) corresponding variation of pair correlation diversion as a function of time; (**d**) final network topology represented by using Delaunay triangulation method and Voronoi diagram.

Figure 2 presents one perfect deployment case by using VFA-SF. At the beginning, all sensors are randomly deployed around the center of a target area, and the initial distribution area $S_{init}=0.7S_{perf}$ [11], as illustrated in Figure 2a. The red dot represents the sensor node, and the blue circle represents the sensing radius. Once the computation based on the algorithm completed, the randomly deployed sensors became organized, which yields good network and coverage, as shown in Figure 2b. Figure 2c shows the corresponding variation of pair correlation diversion in this case. The PCD value drops from 0.91 quickly and finally decrease to 0, which means that the VFA-SF has very good convergence rate and the final distribution should be a perfect hexagon structure. In order to visually compare the difference between the final deployment and the perfect hexagon topology, the Voronoi diagram [19,20] and the Delaunay triangulation [21,22] for the final network configuration were employed, which are illustrated in Figure 2d. The green lines clear show the triangular tessellation generated by Delaunay triangulation, and the blue lines generated by Voronoi diagram present the network structure. The similarity with regular hexagon topology is obvious (more intuitively). In summary, Figure 2 demonstrates the validation of the original virtual spring force algorithm.

**Figure 3:**
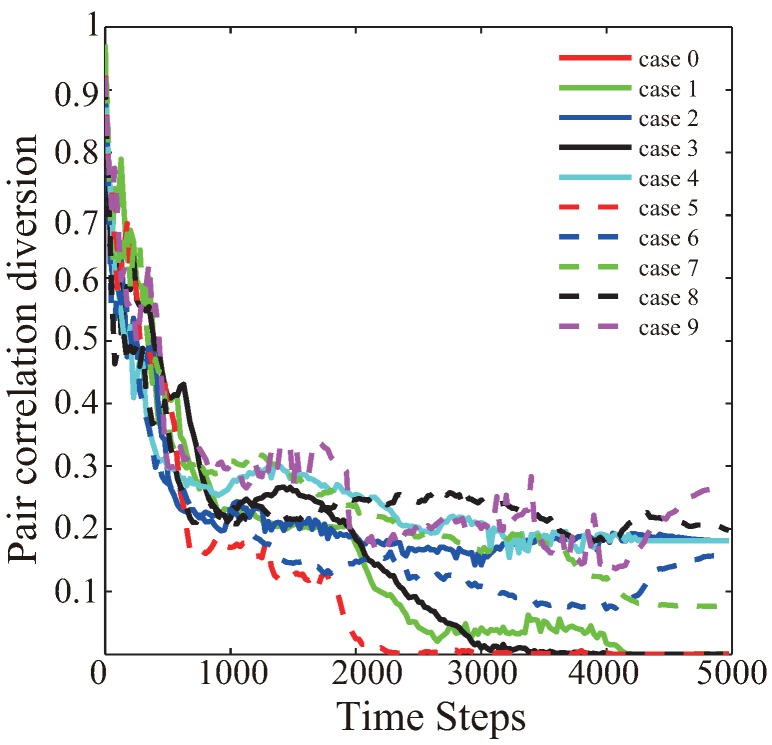
The pair correlation diversion as a function of time from ten independent initial distributions.

In order to evaluate the detailed effect of this VFA-SF algorithm, ten random cases with different initial distributions are independently checked, and the corresponding variations of PCD values as a function of time are shown in Figure 3. It is very clear that different initial sensor distribution can cause different final deployment results despite all the parameters involved in the simulation being identical. Their convergence time also depends on the initial distributions. It appears that the final deployments are still unstable and the PCD value somehow varies depending on the computation time.

**Figure 4:**
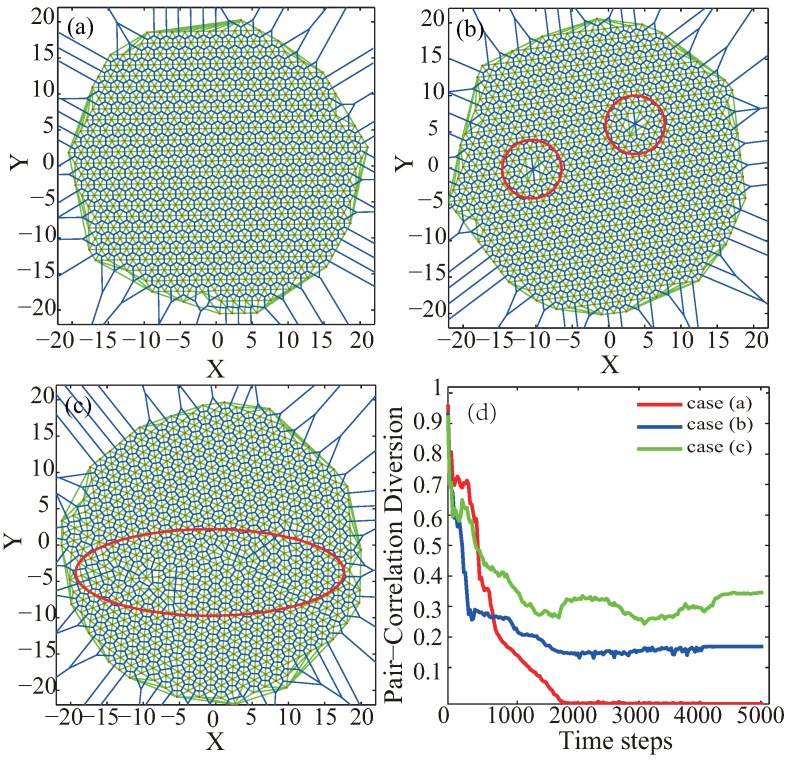
Three typical deployment results for final sensor network topology from VFA-SF: (**a**) perfect network to regular hexagon topology; (**b**) final network with hole marked by red circles; (**c**) final network with some twist balance marked by red ellipse; (**d**) corresponding PCD value of these three simulations experiments.

Based on our experiments, the deployment effect of the final sensor network could be classified into three categories, as illustrated in Figure 4. When the final deployment is perfect to regular hexagonal topology, like Figure 4a, the PCD value converges quickly and is below 0.05; when the final deployment with a hole or node missing, like Figure 4b, the the PCD value converges but still varies between 0.05 and 0.2; when the final deployment with some twisted balance, like Figure 4c, the PCD value is above 0.2.

**Figure 5:**
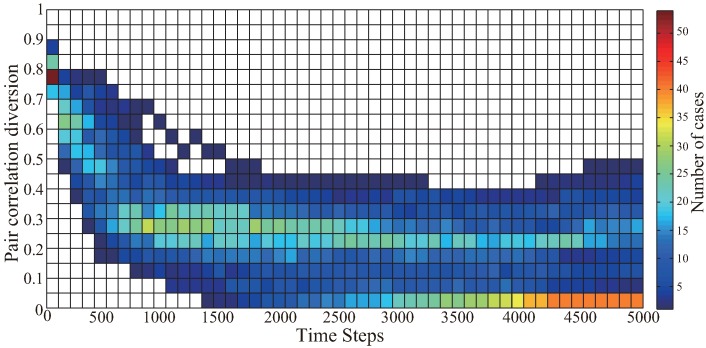
2D contour plot of PCD values as a function of time step for 100 independent simulation experiments of VFA-SF.

Furthermore, in order to statistically test the stability of the original VFA-SF algorithm, 100 simulation experiments have been carried out. The 2D contour plot of the PCD values of these 100 cases is shown in Figure 5. Clearly, the original VFA-SF has poor stability after deploying all sensors to a final configuration. Most of the cases can converge quickly but some of them still have fluctuating PCD value above 0.1. The detailed network topology during the VFA-SF process for every case is analyzed. The numbers of cases for different range of PCD value at the 5000th time step are listed in Table 1.

There are 40 cases with final PCD values between 0–0.05. 20 experiments have been deployed to perfect hexagonal network and the PCD values are 0. Another 20 experiments with the PCD value close to 0.05 can obtain very good hexagonal deployment in most central area of the target region (within the radius =19) but only had somewhat lacking at the edge of the sensor network. That means VFA-SF has nearly 40% possibility to achieve the perfect hexagonal topology.

As for another 60% possibility with PCD value above 0.05, the sensor network topology always has some holes or twisted balance during the deployment process. A noticeable issue is that the PCD value is related to the location of non-regular hexagon topology. If the imperfect hexagonal structure (hole or twisted cell) is closer to the center of the target region, the PCD value will be larger. Instead, if the imperfect deployment is closer to the edge of the whole sensor network, the PCD value will be smaller.

## 4. Further Optimization and Simulation Results

In this section, the VFA-SF algorithm is further optimized to achieve stable node deployment into perfect hexagonal topology, which is denoted as VFA-SF-OPT. The detailed simulation results are shown as verifications.

### 4.1. Optimization Strategy

In the original VFA-SF, the self-deployment process calculates the spring forces, positions and movements for all nodes at each time step. It may lead to the prior formation of a stable hexagonal structure in some subregions, but these subregion can still connect with sensors without exact number as a hexagon so that the deployment results in a hollow or distorted balance. It is difficult to avoid such situation while the algorithm is applied in a large spatial scale.

Since the node deployment in central region is more important than that near the edge, an optimized VFA-SF strategy is proposed here, in which the process begins from the central region. Other sensor nodes in the external region are gradually added to the deployment calculation. At the early stage of deployment, we add an external force $F_{extern}$, whose direction points to the center (0,0), to the most peripheral nodes of the wireless sensor network. It will promote the formation of perfect hexagon topology and effectively avoid holes or twisted balance.

The external force should have the following characteristics: (1) it is always applied to the outermost node of the sensors participating in the deployment algorithm; (2) due to the spring effect, the external forces acting on the most marginal node will be transmitted to the internal network; (3) similar to the centripetal force, external forces should be released when the network deployment is to be completed.

**Figure 6:**
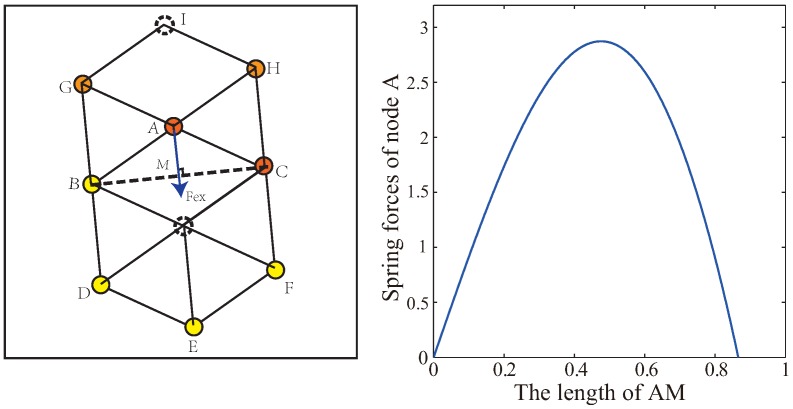
Spring force of sensor node A affected by the length of AM.

As shown in Figure 6, because of the shielding rule within the communication radius $R_{c}$, sensors A, B, C, D, E, F will reach a hexagonal structure only under the effect of VFA-SF. In order to calculate the critical value of external force for node A to break this equilibrium state, it is assumed that node A is the farthest node from the center region and other nodes are fixed. Because the external force only acts on the marginal nodes, the maximum spring resistance of node A comes from nodes A, B, H, and G. The parameters are consistent with the previous section: $D_{m}=\sqrt{3}$, κ=15. An external force is added to make node A move along the line AM so that node A obtains a total spring forces affected by the length of AM, as illustrator in Figure 6.

While the length of AM is 0.477, the spring resultant force of the node A is the largest and the spring force is 2.873 N. In fact, the direction of AM may not point to the center of the network, nevertheless, this external force always points to the center. Therefore, the critical value of external force which is able to break this balance should be larger than that of 2.873. In this section, we set the external force $F_{extern}=3.5$ N. It should be realized that it is a theoretical value, which could help to avoid holes without affecting the formation of regular hexagonal topology.

### 4.2. Simulation Results of Optimized VFA-SF-OPT Algorithm

According to the statistical analysis of basic VFA-SF, the PCD value can generally decrease to ∼0.4 around the 800th time step and corresponding sensor network is going to form an approximate hexagonal topology. So the distribution from VFA-SF at the 800th time step was chosen as the initial distribution of VFA-SF-OPT to go further optimization.

**Figure 7:**
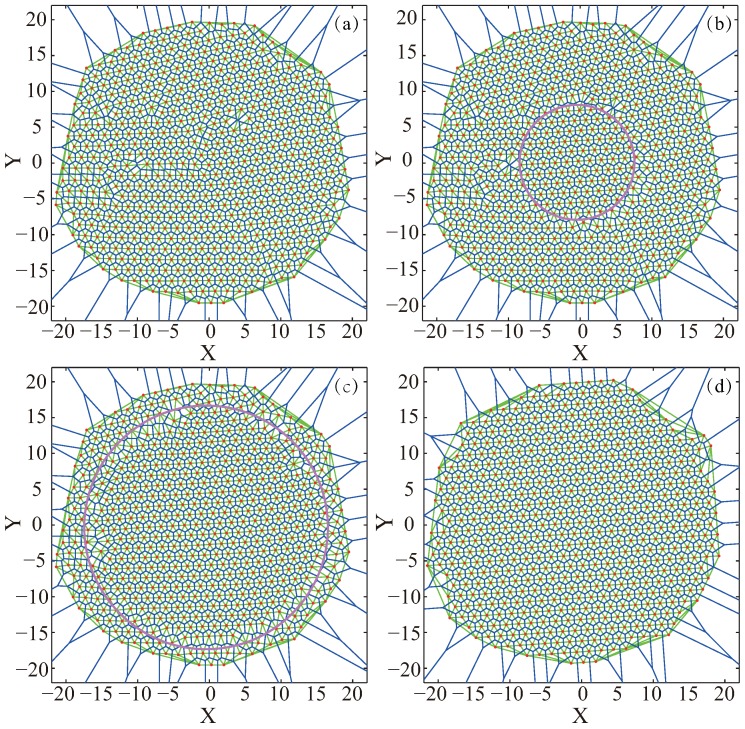
The node distribution in VFA-SF-OPT process: (**a**) initial distribution from VFA-SF at the 800th time step; (**b**) the distribution from VFA-SF-OPT at the 600th time step where only the nodes in the pink circle participated in this optimization algorithm, as time goes on, more and more sensor nodes are involved in the deployment algorithm until they are all involved. (**c**) the distribution from VFA-SF-OPT at the 1200th step; (**d**) final distribution from VFA-SF-OPT.

Figure 7 shows the detailed deployment results during the VFA-SF-OPT process. Figure 7a is the initial distribution at the 800th result of VFA-SF, which is already approximate to a hexagonal structure. Therefore, in the early stage of VFA-SF-OPT, only those sensors close to the center, like the pink circle region in Figure 7b, were involved in the optimized algorithm and successfully archived a perfect hexagonal topology. However, the network formed by other sensors outside of the pink circle is still twisted and has a clear gap with the sensors in the pink circle. As the calculation moves on, more and more sensor nodes are involved in this VFA-SF-OPT algorithm, as shown in Figure 7c. Finally, all sensors are self-deployed into a perfect hexagonal network, as illustrated in Figure 7d.

**Figure 8:**
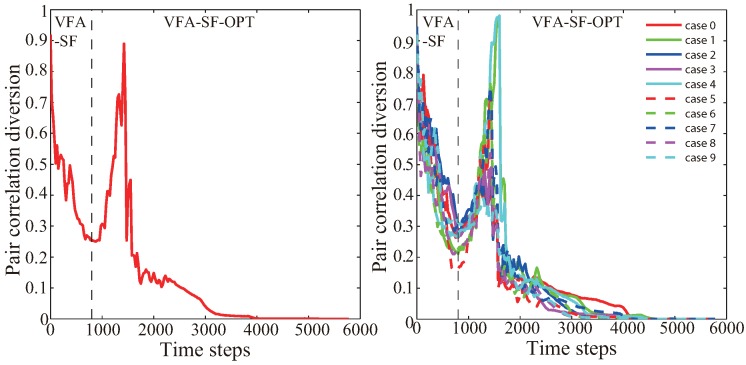
The variation of pair correlation diversion corresponding to the case in Figure 7. The dashed line indicates the 800th time step of VFA-SF.

In addition, Figure 8 presents the variation of pair correlation diversion corresponding to the case in Figure 7, while the dashed line indicates the 800th time step of VFA-SF. The left region clearly demonstrates that VFA-SF can self-deploy the whole sensor network into a good topology as discussed in Section 2. And then, the VFA-SF-OPT takes charge of the main process. At the early stage, the PCD value goes higher since we calculate PCD by using all sensors’ information but only those nodes close to the center region are perfectly deployed into a small hexagonal topology. There is still a gap between the sensors involved in VFA-SF-OPT calculation and those not involved. Usually, those sensors outside of the gap have coverage holes or twisted structure. Once all sensors are participating in the VFA-SF-OPT, the corresponding PCD value sharply decreases to 0.1 and finally becomes 0, which denotes a perfect hexagonal network. Figure 8 shows ten independent experiments with different initial distribution. All PCD values successfully decrease to 0 and the final results are very stable.

**Figure 9:**
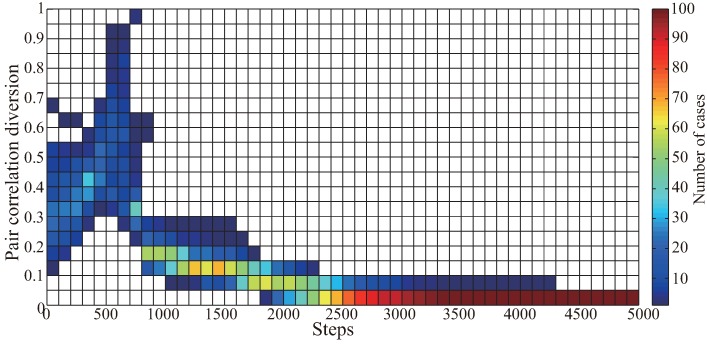
The 2D contour plot for the PCD variation only from VFA-SF-OPT as a function of time step, for 100 cases used in Figure 5.

In order to further verify the effectiveness of the optimization algorithm, the previous 100 simulation experiments as the basic database were retested by the VFA-SF-OPT to start optimization from the 800th time step. Figure 9 shows the 2D contour plot for the PCD variation only from VFA-SF-OPT as a function of time step. All the PCD of those 100 optimization experiments successfully reached the values below 0.05. It verifies that our optimized algorithm is very effective. In other words, the sensor network is capable to be self-deployed into a nearly perfect hexagonal topology regardless of its initial distribution.

**Figure 10:**
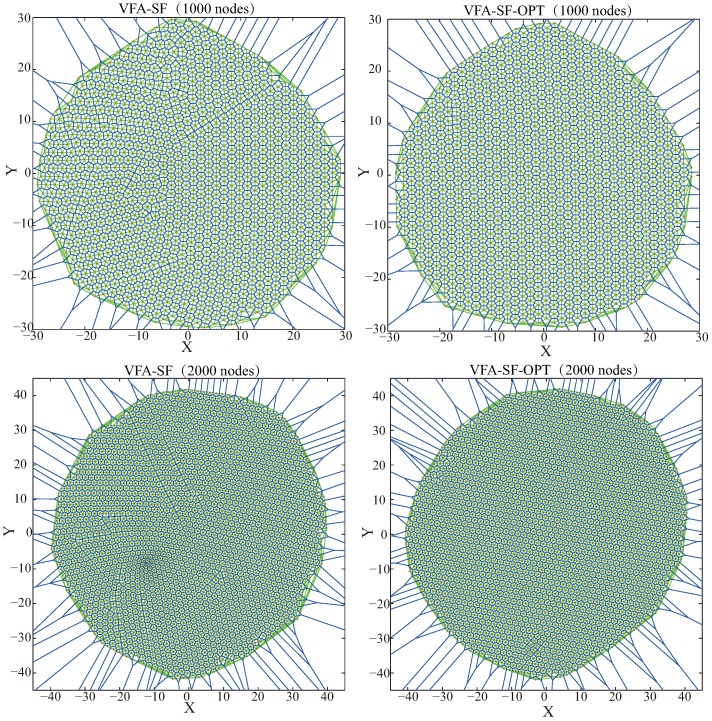
The final network distributions for 1000 sensor nodes (**top**) and 2000 nodes (**bottom**) from VFA-SF (**left**) and VFA-SF-OPT (**right**), respectively.

In addition, Figure 10 presents another two cases with 1000 nodes and 2000 nodes, respectively. Both VFA-SF and VFA-SF-OPT can work for node deployment in a larger WSN. Furthermore, the VFA-SF-OPT clearly has better hexagonal topology in final distribution after zooming in the panels.

### 4.3. Energy Consumption

**Figure 11:**
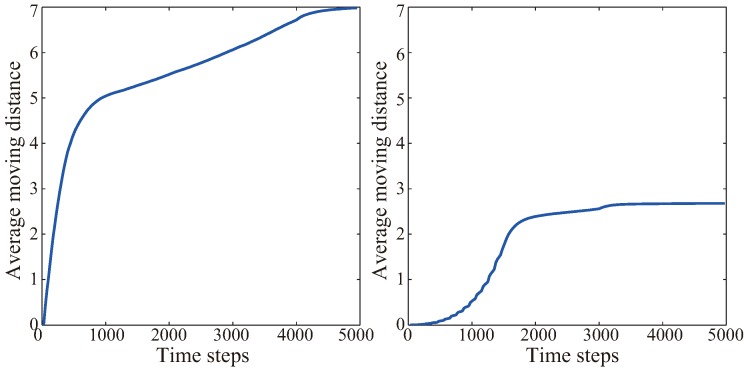
Moving distance from VFA-SF (**left**) and VFA-SF-OPT (**right**).

The comparison of system energy consumption between VFA-SF and VFA-SF-OPT is also an important index in real WSN applications. Here, the average moving distance from all nodes depending on the time step is calculated. The variations of two algorithms as a function of time step are shown in Figure 11. Table 2 lists the detailed maximum distance, minimum distance, average distance and mean square error of one sensor node in VFA-SF and VFA-SF-OPT for different time steps. All sensors move more distance at the early stage of both two algorithms since they need adjust their locations from random places to the appropriate positions. The VFA-SF algorithm always calculates the spring force and drives the node movement for all sensors at each time step so that its moving distance accumulates quickly and will cost more systematic energy consumption. On the contrary, the VFA-SF-OPT only calculates the spring force from the selected sensors in the central region so that the total movement distance gradually increases when more and more sensors involved in this optimization process. It is very clear that VFA-SF-OPT has much better effect in energy consumption since its maximum, minimum and average moving distance are smaller than VFA-SF’s estimations. Based on the comparison in Figure 11 and Table 2, it could be concluded that the VFA-SF-OPT save more system energy than the VFA-SF.

## 5. Conclusions

In this paper, a virtual force algorithm inspired by spring force and a corresponding performance evaluation method for sensor node deployment strategy in a large-scale WSN application is proposed. The simulation experiments clearly demonstrate its validation. However, the statistical analysis still shows that only 40% of cases can lead to a perfect hexagonal network topology. For some initial node distributions, there are still holes or twisted structures in the final sensor network. The VFA-SF lacks stability.

Therefore, we introduced an external force to the VFA-SF and optimize the calculation process of virtual spring force to obtain the VFA-SF-OPT. The node deployment at the 800th time step from VFA-SF was chosen as an initial distribution of the VFA-SF-OPT, which begins to deploy sensors from the center of the target region while other sensor nodes are gradually involved in this optimization strategy. It turns out that the shift from global computation and deployment to centrally preferred deployment is very effective.

Further statistical analysis also showed that all the wireless sensor networks, no matter what the initial distribution is, could achieve a PCD below 0.05, which finally yields perfect hexagonal topology. The optimized algorithm is very stable and effective. In addition, the analysis indicates that the VFA-SF-OPT yields lower energy consumption than the original VFA-SF.

Our two algorithms only concentrates on the deployment strategy for WSN so that the sensor communication and data transmission have not been considered. The VFA-SF formula are simple to be achieved in software and repeated in the experiments after the sensor collects the positions. The algorithms can quickly obtain the coordinates, speed and acceleration information of all nodes. Then, the PCD performance evaluation is carried out every 5 time steps. As for the VFA-SF-OPT, it still has simple formula which only bring in an external central force with respect to VFA-SF. Generally, our algorithms have relatively low complexity.

Moreover, the simulation experiments revealed the potential that the VFA-SF-OPT redeployment can work for non-standard deployment cases. For example, it can start from the 100th or 500th time step of VFA-SF. This means the users can combine this optimization strategy with another kinds of virtual force algorithms to merge the advantages of two different virtual force algorithms. In some real applications, the experiment does not need to calculate 5000 time steps and the PCD performance can be estimated every 20 or 40 time steps. How to further reduce the algorithm complexity or avoid obstacles is definitely a potential interesting topic for our future work.

Our strategy gives a concept of node deployment solution for a large scale WSN. The sensor communication will affect the performance and reduce the effect of the final hexagonal topology. Therefore, in real filed tests, each sensor needs to have a high precision GPS to detect its location. After one sensor approaches its estimated position, it also should wait for a while to receive the updating information from the neighbor nodes. This may cause some time delay for the whole process. Besides, our optimized method is a theoretical analysis which provides a hexagonal sensor network with the maximum coverage area and the minimum number of sensor nodes in the target area. Therefore, the accuracy of node location is important to avoid the possible coverage hole from a sensor’s location error and save system consumption (node number or energy cost) as much as we can. If the high location accuracy can not be satisfied, it can also be solved by slightly decreasing the fixed distance $\sqrt{3}R_{s}$ (for a perfect hexagonal topology). However, it will reduce the whole coverage area and cause some redundancy. We believe that, by choosing suitable algorithm parameters depending on the different environment requirements or application scenarios, users can further reduce the computation complexity and cut down the energy consumption for the whole system. It will probably help engineers to effectively deploy a large number of wireless sensors in the coming future.

## Figures and Tables

**Table 1:** The statistical analysis of final PCD value from 100 independent experiments.

PCD Value	0∼0.05	0.05∼0.1	0.1∼0.15	0.15∼0.2	0.2∼0.25	025∼0.3	0.3∼0.35	>0.35
Number of cases	40	5	5	14	6	18	6	6

**Table 2:** The maximum distance, minimum distance, average distance and mean square error of one sensor node in VFA-SF and VFA-SF-OPT for different time steps.

Algorithm	Time Steps	Max. Dist.	Min. Dist.	Ave. Dist.	Mean Error
VFA-SF	0–5000	10.6086	5.0186	7.0081	1.1784
VFA-SF	0–800	8.6336	2.9020	5.4846	1.1939
VFA-SF	800–1600	1.6464	0.1735	0.4947	0.1707
VFA-SF	1600–2400	0.9941	0.1747	0.4104	0.1373
VFA-SF	2400–3200	1.0669	0.1637	0.4703	0.1370
VFA-SF	3200–4000	1.0382	0.1418	0.5533	0.1818
VFA-SF-OPT	0–4200	5.4899	1.4948	2.6038	0.5719
VFA-SF + VFA-SF-OPT	800 + 4200	11.7351	5.0064	8.0883	1.3212
VFA-SF-OPT	0–5000	5.5319	1.5424	2.6454	0.5740
VFA-SF + VFA-SF-OPT	800 + 5000	11.7858	5.0172	8.1300	1.3226
